# P26 Assessment of fluoroquinolone prescribing in an acute teaching hospital NHS trust

**DOI:** 10.1093/jacamr/dlag102.032

**Published:** 2026-06-26

**Authors:** Callum Sloan, Alina Ceapraga, Ioana-Malina Palistan, Yasmin Elmasry

**Affiliations:** University Hospitals Plymouth NHS Trust, Plymouth, UK; University Hospitals Plymouth NHS Trust, Plymouth, UK; University Hospitals Plymouth NHS Trust, Plymouth, UK; University Hospitals Plymouth NHS Trust, Plymouth, UK

## Abstract

**Background:**

Fluoroquinolones are broad spectrum antibiotics used to manage a range of bacterial infections. Recent regulatory warnings have prompted a review of their use due to the risk of significant adverse effects such as tendonitis and suicidal ideation. Locally, fluoroquinolone prescribing is high compared to other secondary care teaching hospitals within the UK as they are commonly used as alternative treatment options in patients with penicillin allergies.

**Objectives:**

To explore appropriateness of fluoroquinolone prescribing in a secondary care acute teaching hospital at baseline and following interventions to reduce prescribing.

**Methods:**

Data for a cohort of 94 patients prescribed fluoroquinolones in December 2024 was retrospectively extracted to assess baseline prescribing. This included starting specialty, allergy status, and indication, as well as whether the patient was in a high-risk group for fluoroquinolone adverse effects. Risk factors included age over 65 years, renal impairment, solid organ transplantation, concomitant steroids, cardiac risk factors, psychosis/depression, concomitant non-steroidal anti-inflammatory drugs (NSAIDs), or a history of seizures. A national shortage of levofloxacin in May 2025 prompted a review of fluoroquinolone prescribing in the trust and an increase in challenging the use of fluoroquinolones. Interventions included updating respiratory antimicrobial guidelines with options for non-severe penicillin allergies and clinical pharmacists challenging errant penicillin allergies and use of quinolones for inappropriate indications. Follow-up retrospective audits were conducted in July 2025 (*n*=43 patients) and December 2025 (*n*=48 patients) to assess intervention impact. This project was registered with the trust’s Quality Improvement team and did not require ethics approval.

**Results:**

At baseline (December 2024), 54 patients (57%) received fluoroquinolones for appropriate indications, of which 45 (83%) had risk factors for adverse effects. Respiratory (61%) and urinary tract (14%) infections were the most common indications to initiate fluoroquinolones. Post-intervention, initial reaudit (July 2025) found 36 patients (86%) received fluoroquinolones for appropriate indications, with 32 of these patients (88%) having risk factors for fluoroquinolone adverse effects. The most common indications were again respiratory infections (18.6%), as well as urinary, skin, and bone and joint (14% each). Subsequent reaudit in December 2025 found 34 patients (71%) received fluoroquinolones appropriately, with 27 (79%) of these patients having risk factors for fluoroquinolone adverse effects. Respiratory (33%) and gastrointestinal (21%) were the most frequent indication categories. Overall, a substantial proportion of patients with risk factors for adverse effects were consistently prescribed fluoroquinolones regardless of appropriateness (December 2024 85%; July 2025 91%; December 2025 81%). Age over 65 years and renal impairment were consistently the most common risk factors for adverse events identified across the three timepoints.

**Conclusions:**

Fluoroquinolone prescribing for appropriate indications was increased and sustained compared to baseline following interventions to challenge inappropriate use and guideline reviews. Regardless of prescription appropriateness, a substantial proportion of patients with risk factors for adverse effects received fluoroquinolones. Future work will audit prescribers’ awareness of fluoroquinolone adverse effects.

